# Integrative structural and dynamics studies of epidermal growth factor receptor (EGFR)

**DOI:** 10.1038/s12276-026-01744-w

**Published:** 2026-06-05

**Authors:** Juwon Yoon, Sang-Hyeok Lee, Soohan Moon, Harerta Gebremedhn, Teklab Gebregiworgis, Ki-Young Lee

**Affiliations:** 1 School of Pharmacy, Sungkyunkwan University, Suwon, Republic of Korea; 2 Department of Biochemistry, Schulich School of Medicine and Dentistry, University of Western Ontario, London, Ontario Canada; 3 Department of Oncology, Schulich School of Medicine and Dentistry, University of Western Ontario, London, Ontario Canada

**Keywords:** Protein aggregation, Protein aggregation

## Abstract

The epidermal growth factor receptor (EGFR) is one of the most extensively studied targets in cancer therapy, yet its complex regulatory mechanisms remain partially understood. While traditional structural approaches have provided invaluable insights, the static crystal structures of individual domains have been limited to capturing the full dynamic nature of EGFR regulation. Understanding the complete structure of full-length EGFR has been challenging due to the inherent flexibility and complexity of the multidomain membrane protein. Here we integrate recent structural studies using cryo-electron microscopy, molecular dynamics simulations and single-molecule biophysics. Integrated knowledge has revealed that EGFR samples multiple transient conformational states of monomer and higher-order oligomers, and that activation requires coordinated structural transitions between the extracellular domain, transmembrane helix, juxtamembrane region and asymmetric kinase dimer. The allosteric coupling and oligomerization propensity of EGFR are altered by oncogenic mutations, membrane environments and ligand binding. Strategies that incorporate conformational dynamics, allosteric regulation and static structures of EGFR may yield inhibitors with improved selectivity and reduced resistance.

## Dynamic conformational states drive EGFR activation mechanisms

The epidermal growth factor receptor (EGFR), also known as ErbB1, is a key player in cellular signaling, particularly in non-small cell lung cancer. This study delves into the structural and functional dynamics of EGFR, focusing on its role in cancer progression and therapeutic resistance. Researchers used advanced techniques such as cryo-electron microscopy, fluorescence spectroscopy and molecular dynamics simulations to explore EGFR’s conformational states and allosteric mechanisms. They discovered that oncogenic mutations, such as L858R and T790M, enhance EGFR dimerization and oligomerization, leading to ligand-independent activation and increased signaling. These findings are compelling as they provide insights into the molecular basis of drug resistance and suggest new therapeutic strategies targeting EGFR’s structural dynamics. The study highlights the potential for developing more precise treatments for EGFR-mutant cancers, paving the way for personalized medicine approaches.

This summary was initially drafted using artificial intelligence, then revised and fact-checked by the author.

## Introduction

Epidermal growth factor receptor (EGFR), known as ErbB1, is the prototypical member of the ErbB receptor tyrosine kinase family, which also includes ErbB-2 (HER2), ErbB-3 (HER3) and ErbB-4 (HER4), and has become a model membrane protein for studying the structural basis of transmembrane signal transduction^1,2^. Due to the fundamental importance in normal cellular physiology and the frequent dysregulation in cancers, particularly in non-small cell lung cancer (NSCLC), EGFR has been a critical research target and a major focus for therapeutic intervention^3^.

**Fig. 1:**
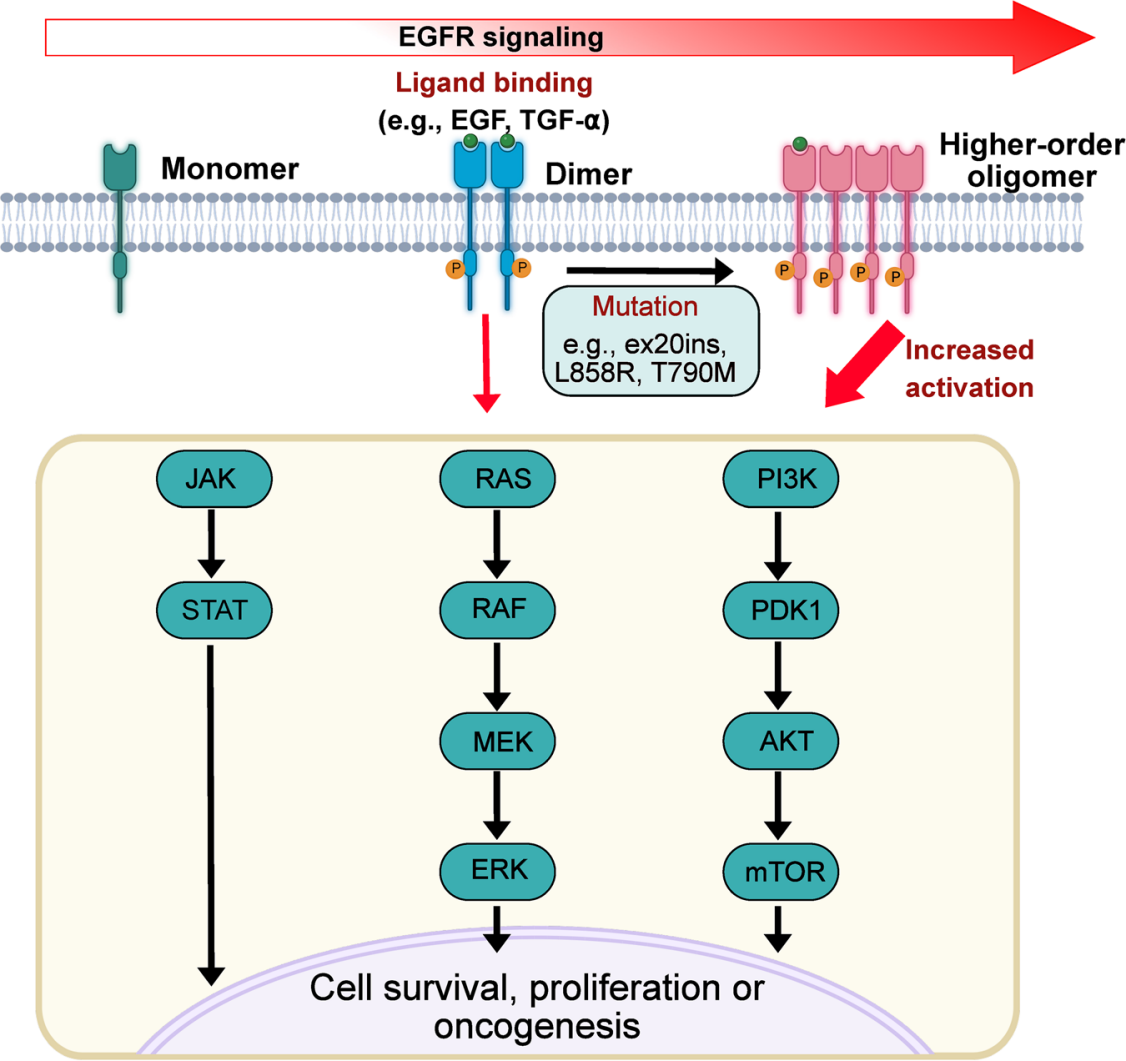
Oligomerization-dependent activation of EGFR. In the absence of ligands, EGFR exists as an inactive monomer, and no intracellular phosphorylation takes place. Upon binding of ligands (for example, EGF and TGF-α) to its ECD, two EGFR monomers dimerize to trigger activation of the ICDs and initiate autophosphorylation of tyrosine residues. EGFR can also form higher-order oligomers, which result in stronger, more sustained downstream signaling. Oncogenic mutations such as L858R (exon 21), exon 20 insertions and T790M (exon 20) promote ligand-independent dimerization and higher-order oligomerization of EGFR and stabilize the active conformation. These alterations enhance phosphorylation signaling even in the absence of ligands. Figure created with BioRender.com (accessed 2 February 2026).

Canonical EGFR signaling begins with ligand-induced extracellular dimerization, followed by asymmetric dimerization of the intracellular kinase domain (KD)^4,5^ (Fig. 1). The dimerization leads to allosteric activation and trans-phosphorylation of tyrosine residues on the receptor’s C-terminal tail and promotes higher-order oligomerization. These sequential processes serve as the regulatory mechanism for enzymatic activation. Some oncogenic mutations and ligands are known to enhance this oligomerization pathway^6,7^. Once activated, EGFR recruits downstream effector proteins or SH2- or PTB-domain-containing proteins to its phosphorylated tyrosines, which propagate intracellular signals and ultimately drive transcriptional changes for cell proliferation, differentiation, apoptosis and angiogenesis^8^.

**Fig. 2:**
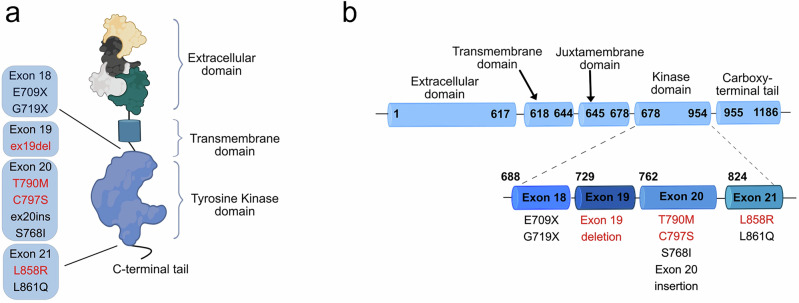
EGFR domain organization and oncogenic mutations. **a** Three structural domains of EGFR. The ECD consists of four subdomains that mediate ligand recognition and receptor dimerization. The TMD is a single α-helical segment connecting the ECD and ICD, which consists of the juxtamembrane domain, tyrosine KD and tyrosine-containing C-terminal tail. **b** Oncogenic mutations occurring within the KD. Oncogenic mutations are indicated below the corresponding exons 18–21 of the KD. These mutations stabilize the active conformation encompassing β3–αC loop, αC-helix, ATP-binding pocket and gatekeeper position in the KD. Common cancer-associated mutations are highlighted in red. Figure created with BioRender.com (accessed 2 February 2026).

The full-length EGFR exhibits a modular architecture consisting of distinct functional domains that are structurally and functionally integrated. The receptor can be divided into three major structural regions: the extracellular domain (ECD), the transmembrane domain (TMD) and the intracellular domain (ICD)^1,9,10^ (Fig. 2a). The ECD (residues 1–617) comprises four subdomains (DI–DIV). DI and DIII constitute the primary ligand-binding units, while DII and DIV are involved in regulatory interactions to promote ligand-dependent dimerization. The TMD (residues 618–644) is a single-pass α-helical segment that spans the plasma membrane and serves as the critical link between extracellular ligand binding and intracellular signaling^11,12^ The ICD encompasses the juxtamembrane domain (residues 645–677), the KD (residues 678–954) and the carboxy-terminal tail (residues 955–1,186)^13^. The ICD is responsible for the enzymatic activity of the receptor and serves as the platform for downstream signaling events^1,14,15^ (Fig. 2b). Notably, oncogenic mutations occurring primarily in the KD stabilize active conformations or increase the affinity for ATP binding^9,16^ (Fig. 2a,b). The ECD, TMD and ICD are tightly interconnected and function as a structural and functional axis that enables EGFR to sense extracellular cues and convert them into intracellular signaling outputs^17^. Therefore, a comprehensive understanding of membrane-associated EGFR requires both static architecture and dynamic interdomain communication.

Binding of ligands such as epidermal growth factor (EGF) or transforming growth factor alpha (TGF-α) to the ECD triggers conformational rearrangements that propagate through the transmembrane helix and juxtamembrane region and stabilize the asymmetric kinase dimer and initiate downstream signaling^18,19^, although some evidence suggests that inactive preformed dimers may also exist^20^. The allosteric coupling is bidirectional^21^. Structural perturbations in the intracellular KD, including conformational shifts induced by phosphorylation, inhibitor binding or oncogenic mutations, can reshape the conformational landscape of the ECD and alter ligand-binding propensity. Recent advances in structural biology techniques, including cryo-electron microscopy and molecular dynamics (MD) simulations, and fluorescence-based techniques, will provide unprecedented insights into the hidden conformational states and allosteric mechanisms of EGFR.

## Oncogenic mutations of EGFR

EGFR mutations are among the most critical genetic alterations in cancer, found in approximately 10–30% of NSCLC patients, with notably higher prevalence in Asian populations (40–55%)^22,23^. The distribution and types of EGFR mutations differ markedly across cancer types, reflecting distinct evolutionary pressures and cellular contexts. Oncogenic EGFR mutations exert structural and functional effects, particularly within the KD and ECD. Notably, mutations frequently found in the KD (Fig. 2a,b) disrupt autoinhibitory conformations, shift the conformational equilibrium toward active states, enhance ATP binding affinity, and promote receptor dimerization and activation^24,25^. Mutations in the ECD can alter ligand-binding specificity, modify dimerization patterns, lead to constitutive receptor activation and reprogram downstream signaling pathways^26^. These alterations drive aberrant EGFR signaling and contribute to cancer progression.

**Fig. 3:**
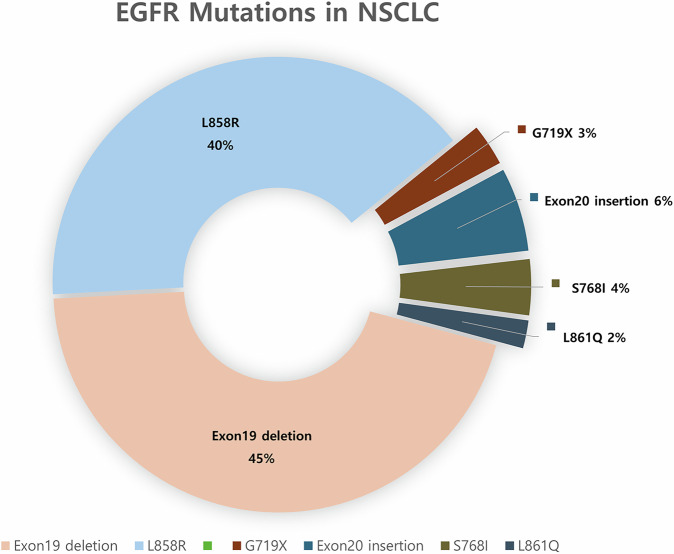
EGFR mutations in NSCLC. The exon 19 deletion is the most prevalent EGFR mutation (~45%), followed by the L858R point mutation (~40%). Less frequent mutations include exon 20 insertions, S768I, G719X and L861Q.

Exon 19 deletions and L858R point mutation account for approximately 85–90% of all EGFR mutations in NSCLC (Fig. 3) and are commonly referred to as ‘classical’ mutations due to their well-characterized sensitivity to EGFR tyrosine kinase inhibitors (TKIs)^22,27,28^. Exon 19 deletions are the most common EGFR mutations in NSCLC, comprising about 44% of all EGFR mutation cases^29–31^. The most common deletion, ΔELREA (removal of E746 to A750), is one of several identified deletions spanning 3 to 18 amino acids. These alterations affect the β3–αC loop region of the KD, stabilizing the αC-helix in its active ‘in’ conformation, increasing ATP binding affinity and kinase activity, and conferring strong sensitivity to first-generation EGFR inhibitors such as gefitinib and erlotinib^32^. The L858R mutation is the second most prevalent EGFR alteration, accounting for approximately 40% of all EGFR mutations^29,30^. This substitution replaces leucine with arginine at position 858 within the activation loop, stabilize the active conformation of the KD. It facilitates new ionic interactions with nearby negatively charged residues and eliminates disordered intermediate states, resulting in a more well-defined conformational landscape^24,33^.

The T790M mutation is the most common mechanism of acquired resistance to first-generation EGFR inhibitors, occurring in approximately 50% of resistant cases^34^. This mutation substitutes methionine for threonine at the gatekeeper position 790, resulting in more than a tenfold increase in ATP binding affinity^35,36^. It significantly reduces the binding affinity of competitive inhibitors and, in some cases, can function as a primary oncogenic driver. The C797S mutation is the primary resistance mechanism to third-generation EGFR inhibitors such as osimertinib^37,38^. It prevents covalent bond formation with irreversible inhibitors, appears in approximately 10% of osimertinib-resistant cases, and may occur in *cis* or *trans* with the T790M mutation, challenging treatment strategies^39,40^.

Uncommon EGFR mutations, although less frequent, have important clinical implications. G719X mutations, present in about 3–4% of EGFR cases, involve substitution of glycine at position 719 with various amino acids and exhibit intermediate sensitivity to TKIs, with an average response rate of 35%^41,42^. Exon 20 insertions, accounting for 4–10% of EGFR mutations, typically occur between amino acids 762 and 774 and involve in-frame insertions of 1–7 amino acids^43^. These alterations often confer resistance to first- and second-generation TKIs owing to their heterogeneous structures; the most common variant, D770-N771insNPG, induces a rigid active conformation^44^. Other uncommon sensitizing mutations include S768I (<5%) and L861Q (~2%), both showing moderate TKI responsiveness and frequently appearing alongside other mutations^45^. In glioblastoma, EGFRvIII, a deletion of exons 2–7, is the most prevalent mutation, found in 25–50% of EGFR-amplified cases^46^. It eliminates most of the ligand-binding domain, resulting in constitutive, ligand-independent signaling. In addition, glioblastoma often harbors ECD mutations such as R84K, A265V and G598V, which modulate ligand recognition, dimerization patterns and receptor activation dynamics^26^.

## ECD structure and dynamics

The ECD of EGFR is composed of four subdomains (DI–DIV) arranged in a modular topology that enables ligand recognition and receptor dimerization^1,11,47^. Domains I and III, which share homologous β-barrel folds, form the primary ligand-binding pocket and engage growth factor ligands through cooperative interactions across both domains^48^. This cooperative binding ensures high-affinity ligand engagement and allows ligands to fine-tune the initial structural transitions of the receptor (PDB ID:1IVO). Domain II is a cysteine-rich region that contains the critical dimerization arm, a β-hairpin structure that mediates receptor–receptor interactions in the activated dimer (PDB ID:1IVO)^49,50^. Domain IV also plays important roles in regulating the conformational state of the receptor and may contribute to receptor dimerization^33^. However, its exact function remains unclear^33^.

**Fig. 4:**
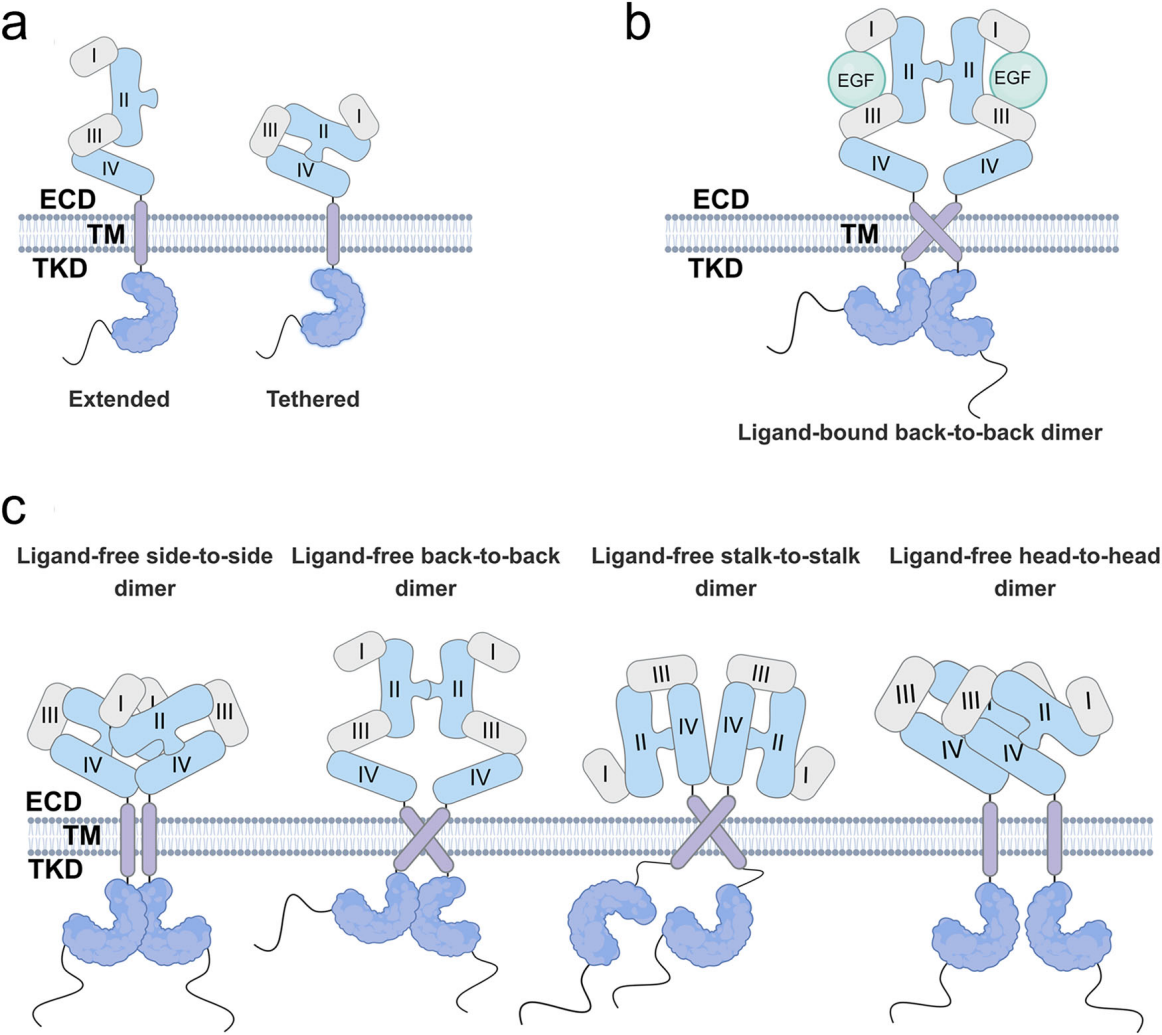
Schematic of the structural models of EGFR monomers and dimers. **a** The EGFR ECD in the extended conformation and the tethered, autoinhibited conformation. **b** A ligand-induced dimer configuration of EGFR, in which the KDs form an asymmetric, active dimer that drives downstream signaling. **c** Ligand-free EGFR dimer configurations. Side-to-side and back-to-back dimers adopt inactive configurations, whereas the stalk-to-stalk dimer is characterized by an asymmetric active kinase dimer, and the ligand-free head-to-head dimer displays no kinase dimerization. Figure created with BioRender.com (accessed 2 February 2026).

Recent cryo-EM studies of full-length EGFR have provided new insights into the role of domain IV in receptor organization and function. These domains undergo conformational changes upon ligand binding, transitioning from a ‘buried’ or ‘tethered’ conformation in the inactive state to an exposed conformation that promotes dimerization^1,51^ (Fig. 4a). EGFR has been proposed to adopt a stable back-to-back dimer conformation of ligand-bound EGFR and to promote downstream signaling (Fig. 4b). However, in the absence of ligands, the ECD samples transient and unstable dimers including side-to-side, back-to-back, stalk-to-stalk and head-to-head conformations incapable of signaling^52^ (Fig. 4c). The ECD interconverts between multiple conformational states relevant to receptor regulation. In the absence of ligand, the domain can exist in a ‘tethered’ conformation where domain II interacts with domain IV (Fig. 4a), potentially serving as an autoinhibitory mechanism^1,53^. However, recent biophysical evidence suggests that this tethered state is highly dynamic and may not represent the predominant conformation under physiological conditions^49^. Ligand binding induces a dramatic conformational change in the ECD, promoting the transition to an ‘extended’ conformation (Fig. 4a) that exposes the dimerization arm and enables receptor dimerization (PDB ID:1IVO). This conformational change involves complex dynamic processes that include local unfolding and refolding events^54^.

MD simulations have revealed that the ECD samples a broad conformational ensemble, with ligand binding shifting the equilibrium toward conformations that are competent for dimerization^47,54^. This conformational selection mechanism provides a molecular explanation for the cooperativity observed in ligand binding and receptor activation. The activation process begins when an EGF-family ligand engages domains I and III of the extracellular region, stabilizing an extended conformation and shifting the receptor away from the partially tethered, autoinhibited ensemble (PDB ID:1IVO)^49^. This ligand-induced stabilization exposes the domain II dimerization arm and reorients the extracellular module relative to the membrane, creating a geometry compatible with receptor dimerization^33^ (Fig. 4b).

The ECD is highly glycosylated, with N-linked glycans comprising approximately 25% of the total mass of the mature receptor^55^. There are ten potential N-glycosylation sites distributed across the four subdomains, with nine of these sites typically occupied by glycan moieties^1^. Glycans may contribute to ECD orientation, ligand-binding affinity and the energetic stability of binding interfaces. Glycans serve as molecular buffers that maintain the spatial relationship between the ECD and the membrane surface, thereby influencing ligand accessibility and dimerization kinetics. They also contribute to ligand-binding affinity by stabilizing the ligand-binding site through noncovalent interactions between glycans and amino acids in the binding region. Altered glycosylation patterns in cancer cells further highlight the functional relevance of this modification. Dysregulated glycan compositions can promote ligand-independent signaling and contribute to therapeutic resistance^56^. Understanding the role of glycosylation in EGFR structure and function is therefore important for both basic biology and therapeutic applications.

## Linking ECD and ICD by TMD

Although EGFR contains only a single TMD α-helix, this segment functions as a critical mechanotransduction element that couples extracellular ligand engagement to intracellular catalytic activation^11,12^. The TMD consists of a single α-helical segment approximately 27 amino acids in length (PDB ID:2N5S)^57^. The transmembrane helix exhibits specific sequence features including the presence of key amino acids that are involved in helix–helix interactions during receptor dimerization.

The TMD helix is responsible for long-range allosteric communication^58^. Upon ligand binding and ECD dimerization, the transmembrane helices undergo specific interactions that are essential for transmitting signal across the membrane (PDB ID:2N5S)^57,59^. These structural rearrangements enable formation of the asymmetric active dimer of intracellular KDs. As a result, the structural and dynamic properties of the TMD may be an emerging allosteric therapeutic target, particularly for modalities such as stapled peptides, lipid-mimetic scaffolds, or conformation-trapping small molecules^58,60^. Some studies have shown that different ligands can induce different transmembrane dimer geometries, which in turn lead to different patterns of ICD activation and downstream signaling^33^. This provides a molecular mechanism for ligand-specific signaling, whereby different growth factors can elicit distinct cellular responses through the same receptor.

## ICD structure and dynamics

### Juxtamembrane domain

The juxtamembrane region couples the transmembrane helix to the intracellular KD and plays an essential role in asymmetric receptor activation. The juxtamembrane domain of EGFR is a 34-amino-acid region that connects the transmembrane helix to the KD (PDB ID:3GOP)^14,61^. This region can be divided into two functional segments: the N-terminal juxtamembrane-A (JM-A) region and the C-terminal juxtamembrane-B (JM-B) region (Fig. 2b). The JM-A region (residues 645–663) contains a polybasic stretch that can interact with negatively charged membrane lipids, particularly phosphatidylinositol-4,5-bisphosphate (PIP2)^60,62,63^. These electrostatic interactions contribute to receptor clustering and lateral membrane organization and promote the formation of an amphipathic helix that stabilizes early dimerization interfaces (PDB ID:3GOP)^14^. By contrast, the JM-B segment (residues 664–677) directly participates in asymmetric kinase dimer formation by acting as a molecular ‘latch’. Through extended contacts with the C-lobe of the partner kinase, JM-B stabilizes the active asymmetric dimer configuration and ensures efficient catalytic activation^61,64^. JM-A and JM-B act as a coordinated structural and regulatory module that integrates membrane context, receptor dimer geometry and kinase activation.

### Kinase domain

The KD represents the catalytic core of EGFR signaling and serves as a key regulatory node controlling receptor activation, substrate phosphorylation and inhibitor sensitivity^1,61,65^. The KD exhibits the typical protein kinase fold, consisting of a smaller N-terminal lobe and a larger C-terminal lobe. The N-lobe contains the ATP-binding cleft and the regulatory αC helix, whereas the C-lobe is predominantly α-helical and has the activation loop and catalytic residues^13^. The KD can adopt multiple conformational states that differ in their catalytic activity^59,66,67^. The active conformation is characterized by specific arrangements of key structural elements, including the αC helix in the ‘in’ position, the activation loop in an open conformation, and the DFG motif in the ‘in’ position^13^. Inactive conformations can involve displacement of these elements, leading to reduced or eliminated catalytic activity. Regulation of these conformational states is mediated primarily through asymmetric dimerization (Fig. 4b,c), in which one KD (the ‘activator’) allosterically stabilizes the active configuration of the partner kinase (the ‘receiver’) via defined protein–protein interface contacts (PDB ID:3GOP)^68^. This mechanism is unique among receptor tyrosine kinases and creates multiple structural checkpoints that can be exploited by oncogenic mutations such as L858R and T790M, or targeted by small-molecule TKIs^65,69^.

### Carboxy-terminal tail

The carboxy-terminal tail of EGFR is a large (>200 amino acids) region that is largely disordered in solution^14,70^. The carboxy-terminal tail represents the primary signaling output interface of EGFR and functions as a flexible regulatory module. Embedded within this flexible architecture are multiple tyrosine residues that serve as autophosphorylation sites and docking motifs for SH2- and PTB-domain-containing adaptor proteins, enabling recruitment of downstream signaling complexes^13,71^. Despite its disordered nature, the C-terminal tail is not structurally random. It adopts preferred conformational states that influence the accessibility of phosphorylation sites and interaction motifs, providing a flexible mechanism for context-dependent regulation^70,72^. Disorder-to-order transitions can occur upon phosphorylation, protein binding or changes in local membrane environment, allowing the tail to couple receptor activation state to pathway selection^73^.

## Membrane-dependent oligomerization of EGFR

A major conceptual shift in understanding full-length EGFR structure has come from the recognition that the receptor forms nanoscale clusters within native membranes. These assemblies are not random aggregates; rather, they represent higher-order structural arrangements that modulate receptor activation efficiency, ligand sensitivity and signaling output^74–76^.

The membrane environment plays a crucial role in the structure and function of full-length EGFR. The receptor is not simply embedded in a uniform lipid bilayer but is organized within specific membrane domains that influence its conformation and activity^77^. Studies using various membrane mimetic systems have shown that the lipid composition and membrane organization can affect EGFR structure and function and that the TMD structure and dynamics are sensitive to the membrane environment (PDB ID:2N5S)^57,60,64^.

The nanoclustering of EGFR has important functional implications. The organization in membrane domains is dynamic and can be influenced by factors such as ligand binding, mutations and cellular conditions^60,77,78^ (Fig. 1). This dynamic organization provides a mechanism for spatially organizing signaling events and controlling the specificity and amplitude of receptor responses. The nanoclustering also provides a mechanism for signal amplification and emergent cooperation. When receptors exist in close proximity, conformational changes and activation events can propagate across neighboring molecules, increasing signaling efficiency and enabling collective activation responses^7^. Through these effects, nanoscale receptor organization becomes a determinant of EGFR signaling capacity.

Cryo-electron tomography studies have provided direct visualization of EGFR clusters in native membrane environments, revealing that receptors form ordered arrays with specific inter-receptor distances and orientations^17,78^. These clusters are formed through a combination of protein–protein contacts and selective interactions with specific membrane lipids, such as PIP₂ and cholesterol-rich domains, highlighting the cooperative role of both structural and membrane-mediated interactions in stabilizing these assemblies^60^.

Emerging biophysical and structural evidence indicates that dimers can extend to higher-order oligomers such as tetramers and larger clusters^7^. These assemblies appear to enhance activation efficiency, enable signaling cooperativity and influence receptor trafficking and signaling duration^73^. The interfaces that stabilize oligomers may form multiple structural layers, including extracellular interfaces, transmembrane crossing angles and kinase–kinase contacts (Fig. 6a), suggesting that higher-order assembly represents an additional regulatory checkpoint.

## Mutation-enhanced oligomerization of EGFR

**Fig. 5:**
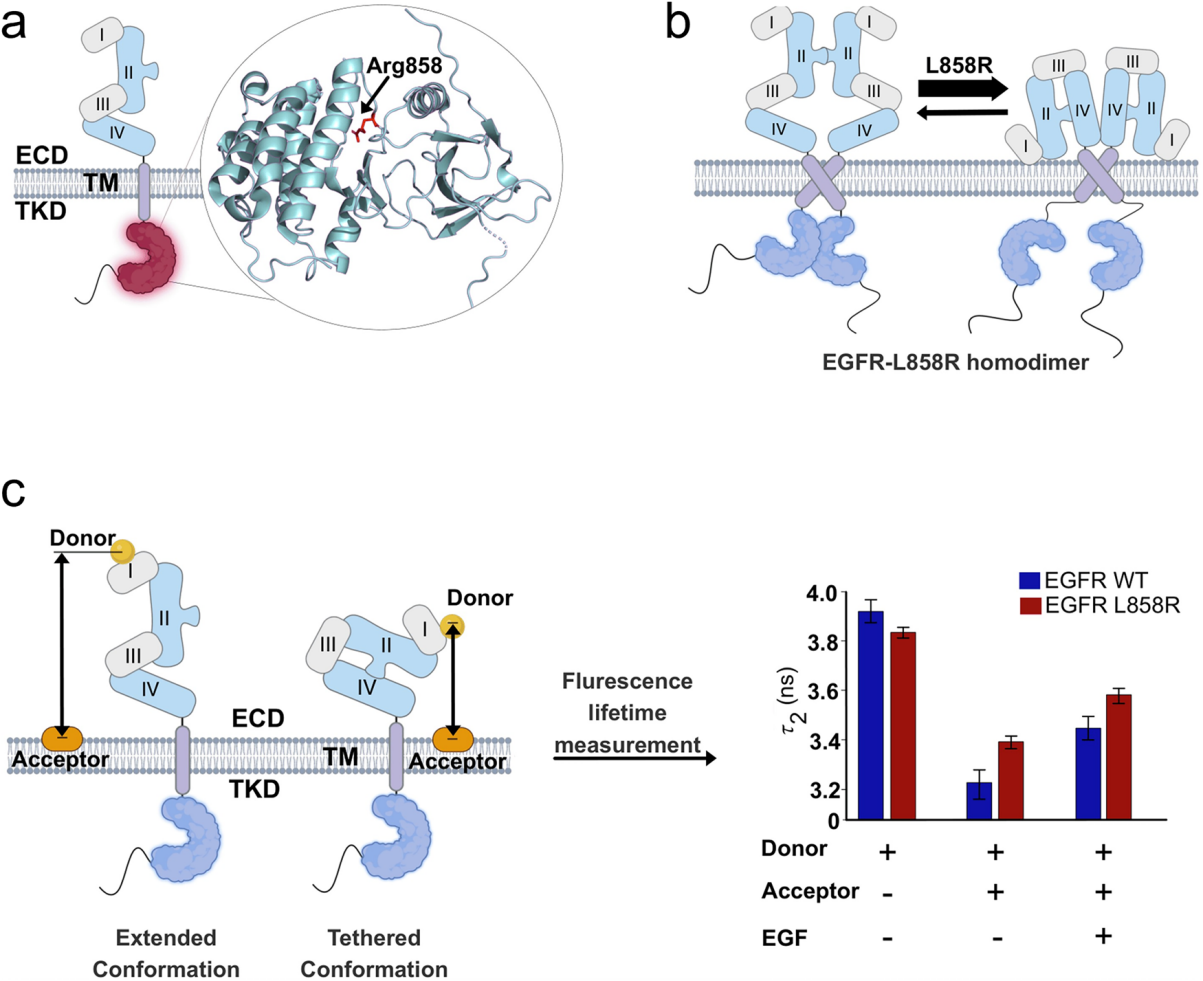
L858R-induced changes in the dynamics and membrane orientation of EGFR. **a** Mapping L858R mutation onto the crystal structure of EGFR-KD (PDB ID: 4LL0). **b** L858R-induced equilibrium shift from the inactive back-to-back ectodomain/head-to-head kinase dimer toward the active stalk-to-stalk ectodomain/asymmetric kinase dimer configuration. This structural model was derived from a combination of MD simulations and advanced fluorescence imaging. **c** FRET–FLIM analysis used to probe the conformational state of the EGFR ECD. On the left, schematic structural models of EGFR in its tethered, autoinhibited state versus its extended conformation are depicted, and the donor fluorophore is fused to the EGFR N terminus, while the acceptor fluorophore is incorporated into the outer leaflet of the plasma membrane. On the right, plots for representative donor fluorescence lifetime of wild-type EGFR versus L858R are shown. Increased donor lifetime of L858R represents a greater donor–acceptor distance and an extended conformation. Figure created with BioRender.com (accessed 2 February 2026).

Previous studies have demonstrated that oncogenic mutations within the EGFR KD, including L858R and T790M, promote receptor dimerization and higher-order oligomerization through multiple mechanisms^5,24,79^. These mutations exert allosteric effects on the ECD and stabilize the dimeric configuration even in the absence of ligand, supporting the existence of structural coupling between the extracellular and intracellular domains. The L858R mutation located within the ICD shifts the ectodomain from a tethered conformation to an extended, dimer‑competent state^80^ (Fig. 5). Förster resonance energy transfer (FRET)-based analyses determined the tethered or extended conformation of the EGFR ectodomain by using donor fluorescence lifetime as an indicator of the distance to the acceptor fluorophore. In ligand-free conditions, wild-type EGFR exhibits a markedly short lifetime, whereas the EGFR L858R mutant displays a lifetime comparable to that of ligand-bound EGFR, supporting the conclusion that the intracellular mutation promotes ligand-independent dimerization. Consistent with this structural model, recent MD simulations and fluorophore localization imaging with photobleaching (FLImP) measurements indicated that EGFR–L858R dimers preferentially adopt an asymmetric kinase dimer configuration^52^ (Fig. 5b). In addition, two-color quantum dot tracking methods were used to quantify dimer off-rates, showing that wild-type EGFR exhibits a rapid dimer dissociation rate under ligand-free conditions, whereas the L858R mutant maintains a markedly slower off-rate comparable to that of ligand-bound receptors, indicative of a more stable dimer^80,81^. These data suggest that the oncogenic mutation promotes a basal conformational state that mimics the active ligand-bound state, thereby driving constitutive signaling activity and higher-order oligomers.

**Fig. 6:**
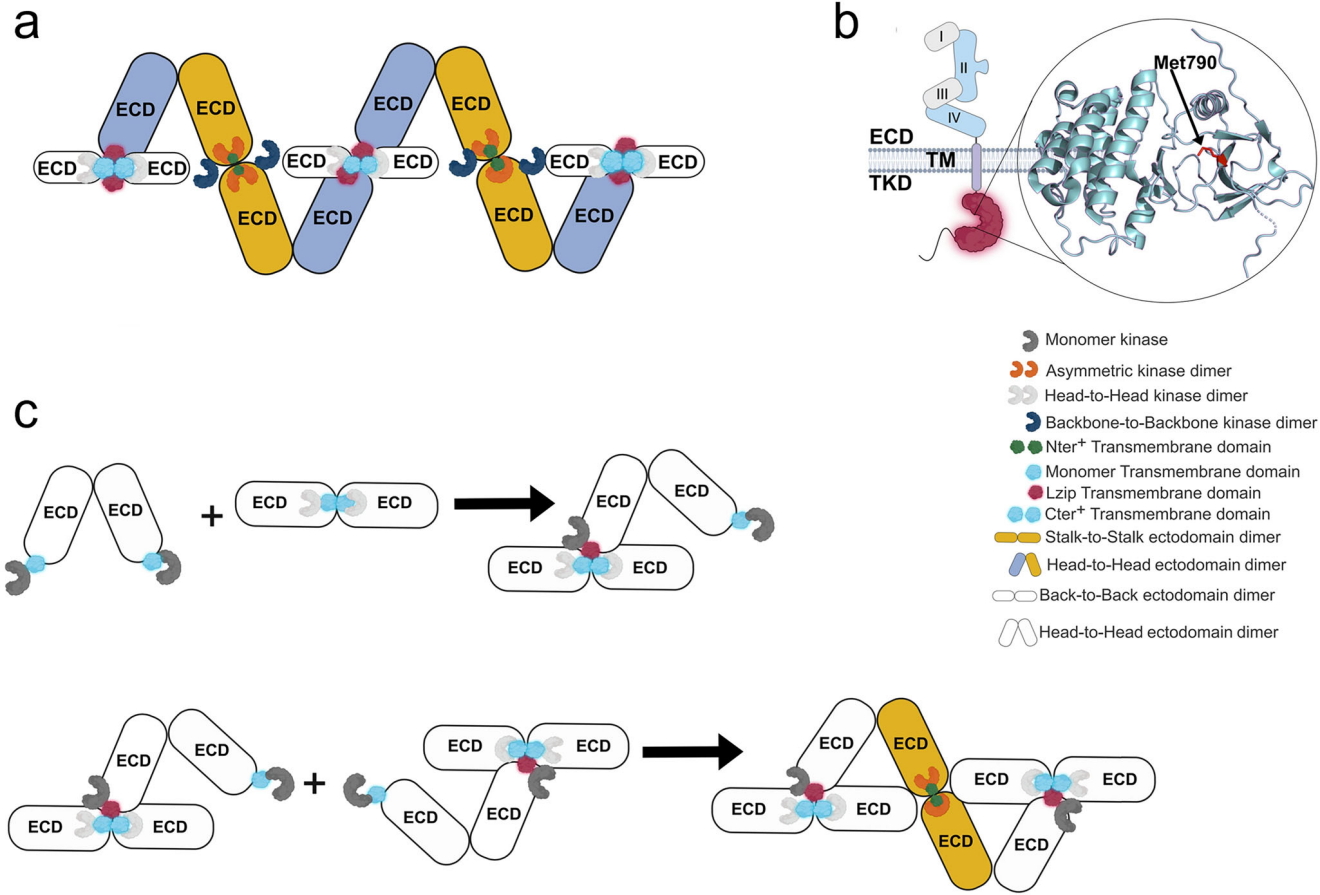
Possible oligomerization interfaces for wild-type EGFR and the T790M mutant. **a** Possible interfaces of ligand-free hetero-conformation oligomers of EGFR. These assemblies are highly unstable and remain limited in size. The back-to-back ectodomain/head-to-head kinase dimer is transient, whereas T790M mutation stabilizes this dimer and promotes higher-order oligomerization. **b** Mapping T790M mutation onto the crystal structure of EGFR-KD (PDB ID: 4LL0). **c** Ligand-free hetero-conformation oligomerization model of EGFR-T790M. A tetramer is assembled from a head-to-head ectodomain/2× kinase monomer subunit and a back-to-back ectodomain/head-to-head kinase dimer subunit via the Lzip transmembrane interface. Two of these head-to-head/back-to-back tetramers further assemble into a stalk-to-stalk ectodomain/asymmetric kinase dimer subunit. Figure created with BioRender.com (accessed 2 February 2026).

Other recent data suggest that EGFR can form a conformational diversity of oligomers in the absence of ligand, and these ligand-free oligomers are stabilized by the T790M mutation^36,82,83^ (Fig. 6b). Specifically, the T790M mutation stabilizes the back-to-back ectodomain/head-to-head kinase dimer through the side-to-side kinase interface, which promotes the formation of larger oligomers without compromising phosphorylation and ultimately enhances cellular growth. The proposed mechanism is that the T790M mutant first stabilizes the back-to-back ectodomain/head-to-head kinase dimer even in the absence of ligand. This configuration sequesters kinase monomers via the side-to-side interface, thereby preventing formation of the stalk-to-stalk ectodomain/asymmetric kinase dimer through the backbone-to-backbone interface (Fig. 6c). The stabilized back-to-back dimer thus serves as a nucleation seed for the assembly of larger and more stable hetero-conformational oligomers. The FLImP data showing lateral pairwise separations (0–70 nm) between EGFR ectodomains were consistent with the live‑cell single‑particle tracking data that EGFR‑T790M exhibits a lower diffusion coefficient and increased clustering compared with wild-type EGFR^36^.

## Structure-targeted drug discovery

Traditional approaches have focused on inhibiting the KD of EGFR. However, recently accumulating data on the allosteric network of the receptor provide additional opportunities for intervention beyond classical ATP-competitive inhibition^3^. Specifically, rather than targeting the KD in isolation, emerging approaches seek to exploit the conformational ensemble, domain coupling mechanisms and dynamic oligomerization to inhibit more selective pathways for EGFR activation, as described below.

### Conformation-selective inhibitors

Small molecules and allosteric TKIs are known to target sites distant from the ATP-binding site, stabilizing inactive conformations or disrupting the asymmetric kinase dimer^84,85^. EAI045 can bind to an allosteric pocket that becomes preferentially accessible due to mutation induced structural perturbations, such as L858R and T790M, resulting in EGFR inactivation^86–88^.

### ECD modulators

Therapeutic antibodies and engineered protein scaffolds targeting the ligand-binding site, domain II dimerization arm or extended extracellular conformations can prevent receptor activation upstream of kinase engagement. Necitumumab is a clinically approved anti-EGFR mAb (PMID: 18275813) that blocks ligand binding and potentially stabilizes inactive conformations^89,90^. Such agents may also bias receptor clustering, ligand cooperativity or membrane spatial organization.

### Modulators of transmembrane and juxtamembrane domains

Lipid-interacting compounds designed to disrupt helix packing, PIP₂-dependent stabilization or juxtamembrane latch formation represent a growing modality that functionally disconnects extracellular signals from intracellular catalytic outcome^12,60,64^.

### Oligomerization modulators

Pathological signaling often depends on altered receptor clustering, higher-order assembly, or lipid-domain partitioning. Therefore, targeting EGFR nanoscale organization offers a way to regulate signaling pathways at the level of receptor oligomerization states. Rationally designed peptides derived from the EGFR dimerization interface can disrupt receptor dimerization and suppress downstream signaling, highlighting receptor–receptor contacts as viable therapeutic targets^7,52,91^.

Advanced understanding of oncogenic EGFR mutations is being driven by several key developments, including sequencing technologies, mutation-specific TKIs, diagnostic tools that are capable of detecting complex mutations, and the implementation of combination therapy strategies that can overcome resistance^92^. Based on these advancements, we believe that more precise and personalized therapeutic approaches for EGFR mutant cancers will be rapidly accelerated.
